# High expression of focal adhesion kinase (p125^FAK^) in node-negative breast cancer is related to overexpression of HER-2/neu and activated Akt kinase but does not predict outcome

**DOI:** 10.1186/bcr977

**Published:** 2005-01-07

**Authors:** Klaus Jürgen Schmitz, Florian Grabellus, Rainer Callies, Friedrich Otterbach, Jeremias Wohlschlaeger, Bodo Levkau, Rainer Kimmig, Kurt Werner Schmid, Hideo Andreas Baba

**Affiliations:** 1 Institute of Pathology, University Hospital of Essen-Duisburg, Essen, Germany; 2 Department of Obstetrics and Gynecology, University Hospital of Essen-Duisburg, Essen, Germany; 3 West German Cancer Centre, Essen, Germany; 4 Institute of Pathophysiology, University Hospital of Essen-Duisburg, Essen, Germany

**Keywords:** Akt, breast cancer, focal adhesion kinase, HER2, prognosis

## Abstract

**Introduction:**

Focal adhesion kinase (FAK) regulates multiple cellular processes including growth, differentiation, adhesion, motility and apoptosis. In breast carcinoma, FAK overexpression has been linked to cancer progression but the prognostic relevance remains unknown. In particular, with regard to lymph node-negative breast cancer it is important to identify high-risk patients who would benefit from further adjuvant therapy.

**Methods:**

We analyzed 162 node-negative breast cancer cases to determine the prognostic relevance of FAK expression, and we investigated the relationship of FAK with major associated signaling pathways (HER2, Src, Akt and extracellular regulated kinases) by immunohistochemistry and western blot analysis.

**Results:**

Elevated FAK expression did not predict patient outcome, in contrast to tumor grading (*P *= 0.005), Akt activation (*P *= 0.0383) and estrogen receptor status (*P *= 0.0033). Significant positive correlations were observed between elevated FAK expression and HER2 overexpression (*P *= 0.001), as well as phospho-Src Tyr-215 (*P *= 0.021) and phospho-Akt (*P *< 0.001), but not with phospho-ERK1/2 (*P *= 0.108). Western blot analysis showed a significant correlation of FAK Tyr-861 activation and HER2 overexpression (*P *= 0.01).

**Conclusions:**

Immunohistochemical detection of FAK expression is of no prognostic significance in node-negative breast cancer but provides evidence that HER2 is involved in tumor malignancy and metastatic ability of breast cancer through a novel signaling pathway participating FAK and Src.

## Introduction

Breast cancer is a major cause of death among women. Adjuvant systemic therapy may considerably improve survival rates, but it is associated with severe toxic side effects. Especially in patients with node-negative breast cancer, the pros and cons of adjuvant systemic therapy are always critically weighted out. The identification of novel markers for node-negative high-risk patients who would benefit from adjuvant therapy is therefore of major importance. The aim of the present study was to assess the prognostic relevance of focal adhesion kinase (FAK) expression in node-negative breast cancer. In addition, the present report was designed to investigate the consequence of FAK expression on two major downstream targets (namely, Akt and Erk1/2) and to elucidate its connection with the HER2 signaling pathway.

The invasion and metastasis of cancer is a complex process including changes in cell adhesion and motility that allow tumor cells to invade and migrate through the extracellular matrix. Some of these alterations may occur at focal adhesions, which are cell/extracellular matrix contact points containing membrane-associated, cytoskeletal and intracellular signaling molecules [1]. The survival of normal epithelial cells critically depends on cell–cell and cell–matrix contact. Without these contacts epithelial cells die through the controlled process of apoptosis, termed *anoikis *[2]. FAK is a tyrosine kinase considered a central molecule in integrin-mediated signaling, and it is involved in cellular motility and protection against apoptosis [3-7]. The importance of FAK in epithelial cell biology is underlined by the findings that epithelial cells lines expressing constitutively active FAK survive in suspension and that cells derived from FAK^-/- ^mouse embryos exhibit reduced migration [8,9].

High levels of FAK have been found in a variety of tumors, including head and neck carcinomas, ovarian carcinomas, thyroid carcinomas and colon carcinomas [10-12]. Only two studies have so far described elevated FAK expression in human breast cancer and preinvasive lesions in contrast to benign breast lesions in small cohorts [13,14]. The prognostic value of FAK expression in breast cancer remains unclear. Regarding other cancer types, three studies demonstrated varying results in colon cancer, squamous cancer and hepatocellular carcinoma [15-17].

Recent studies have identified HER2, a prognostic marker for aggressiveness in breast cancer, to influence the migratory behavior of breast cancer cells *in vitro *through a novel signaling pathway involving phosphorylation of FAK at tyrosine 861 by its upstream kinase c-Src [18,19]. These findings suggest that the Her-2/neu signaling pathway may influence migration and metastasis of breast cancer cells through activating the Src/FAK signaling pathway. There are several downstream targets of FAK such as the Ras-ERK signaling cascade, which is activated by extracellular, frequently mitogenic ligands and results in increased cellular proliferation and malignancy *in vitro *and *in vivo *[20,21]. FAK also impacts on the phosphotidylinositol-3-kinase (PI3K)/Akt-signaling pathway that plays a central role in tumorigenesis [22,23]. In node-negative breast cancer, we have previously shown that activated Akt is a prognostic parameter [24].

The aim of the present study was to assess the prognostic relevance of FAK expression in node-negative breast cancer. In addition, the present report was designed to investigate the correlation of FAK expression with two major downstream signaling pathways, Akt and Erk1/2, and to elucidate its connection with the HER2 signaling pathway.

## Materials and methods

### Patients

This study comprised 162 female breast cancer patients (mean age, 59 years) from the Department of Gynecology, University of Essen-Duisburg, Germany, who underwent surgery and further adjuvant therapy between 1989 and 1996. Complete clinical records and follow-up information were available in all cases. The negative lymph node status was confirmed by axillary dissection. All surgical material was fixed in 4% formalin and routinely processed. The tumors were classified according to the pTNM system (sixth edition) and were graded according to Elston and Ellis [25]. Twenty-seven of the 162 patients died during follow-up, the cause of death being unknown in two cases. Altogether 19 patients died of breast cancer, and six patients were excluded from survival analysis having died from either benign or other cancer diseases. Statistical analysis was based on a mean follow-up period of 7.48 years. Table 1 summarizes the clinicopathological parameters of this study.

### Immunohistochemistry

The primary antibodies used in this study are presented in Table 2. Immunohistochemistry was performed on paraffin sections 5 μm thick. The alkaline phosphatase anti-alkaline phosphatase method was used for antibody demonstration. Antigen retrieval was carried out with 0.01 M citrate buffer at pH 6.1 for 20 min (both phospho-Akt antibodies), for 40 min (phospho-ERK1/2) and for 20 min (FAK, phospho-Src Tyr-215 and phospho-Src Tyr-416) in a hot water bath (95°C). Antibodies were incubated overnight in a humidified chamber at 4°C. Positive controls were included in each staining series. No significant staining was observed in the negative controls using mouse immunoglobin replacing the primary antibody. The DAKO HerceptTest™ (DakoCytomation, Glostrup, Denmark) and a HER2/ErbB2 polyclonal antibody at a 1:50 dilution (rabbit polyclonal antibody; Cell Signaling Technology, Beverly, MA, USA) were used for the detection of HER2 protein expression. The estrogen receptor (ER) status of the tumors was determined using a monoclonal anti-human antibody as previously described [24].

### Evaluation of immunostaining

#### FAK immunostaining

The constant positive staining of smooth muscle cells of tumor vessels served as the positive internal control as previously described [26]. If more than 20% of carcinoma cells in a given specimen were stained more intensely than smooth muscle cells, the sample was classified as strong FAK overexpression (3+). In the case of equal FAK immunostaining compared with that of vascular smooth muscle cells, the sample was classified as intermediate expression (2+). When FAK immunostaining was weaker than that of the internal control, the tumor was classified as low FAK expression (1+). Tumors lacking FAK immunostaining were classified as negative (0). For statistical analysis, negative (0), intermediate (1+ and 2+) and strong (3+) groups were created.

#### Phospho-Akt and phospho-ERK1/2 immunostaining

The number of positive immunoreactive tumor nuclei was counted within 300 cells at the invasive tumor front, and is expressed as a percentage. Cytoplasmatic phospho-Akt staining was noticed but was not part of the scoring system. Tumor cells with easily detectable specific phospho-ERK1/2 immunostaining, independent of the amount of stained cells, were scored as strongly positive (2+). Tumors exhibiting a detectable but faint immunostaining were scored as weak (1+), whereas tumors with a minimal, hardly detectable or missing staining pattern were classified as phospho-ERK1/2-negative (0).

#### Phospho-Src Tyr-215 and phospho-Src Tyr-416 immunostaining

Most tumor samples showed a membranous staining with varying intensity. Four samples revealed a strong cytoplasmatic staining. Nuclear staining was absent. Tumor samples with a readily detectable membranous staining or with a strong cytoplasmatic staining were classified as positive. Samples lacking or with a weak membranous staining or with a weak cytoplasmatic staining were classified as negative.

### HER2 protein and ER status

The Her-2/neu status was determined according to the evaluation system of the HerceptTest™. For statistical analysis, negative (DAKO score 0 and 1+) and positive (DAKO score 2+ and 3+) groups were created. A tumor was regarded as ER-negative if none or less than 10% of the tumor cells showed weak or missing nuclear immunostaining.

### Western blot analysis

Total cell protein of seven representative frozen human breast cancer samples was extracted in lysis buffer and the protein concentration was measured to load equal protein amounts. Each lane consisted of 50 μg protein, and was size fractioned by electrophoresis on 10% polyacrylamide-SDS gels and electrotransferred to a polyvinylidene fluoride membrane using a tank-blotting system. After staining the membranes with reversible Ponceau red solution to confirm equal protein loading, the membranes were blocked with 5% non-fat dry milk in Tris-buffered saline for 1 hour at room temperature. The membranes were then incubated with the anti-HER2/ErbB2 antibody (polyclonal rabbit HER2/erbB2 antibody, 1:1000; Cell Signaling Technology, Inc.), anti-phospho-FAK antibody (Tyr-861, polyclonal goat antibody, 1:50; Santa Cruz Biotechnology, Inc., Santa Cruz, CA, USA) and anti-phospho-Src antibody (Tyr 215, polyclonal rabbit antibody, 1:2000; Sigma-Aldrich, Inc., St Louis, MO, USA) for 16 hours at room temperature. After incubating the membrane for 1 hour with goat anti-rabbit/donkey anti-goat antibody conjugated to horseradish peroxidase, the antigen–antibody complex was visualized by chemiluminescence (Amersham Biosciences, Piscataway, NJ, USA).

### Statistical analysis

FAK, phospho-Erk1/2, Her-2/neu, phosph-Src Tyr-215/416 and ER immunostainings were assessed by a set of pathologists (KJS, FG, JWO, FO) in a blind-trial fashion without knowledge of the clinical outcome. In cases of disagreement, the slides were evaluated by two pathologists and a final decision was made. Two of the authors assessed the percentage of phospho-AKT-positive tumor cells and the mean percentage of both counts was used for statistical analysis. Staining scores of both phospho-Akt antibodies (Santa Cruz Biotechnology Inc. and Cell Signaling Technology Inc.) revealed a high, significant interobserver concordance (Pearson's *r *= 0.671, *P *< 0.001).

Results obtained from phospho-Akt immunostaining performed with the antibody from Santa Cruz showed less background staining, and were therefore used for statistical analysis. All data were converted to a PC and statistically analyzed using SPSS Version 10 for Windows (SPSS Inc., Chicago, IL, USA). Relationships between ordinal parameters were investigated using two-tailed chi-squared analysis (or Fisher's exact test where patient numbers were small). The relationship between FAK expression and the percentage of phospho-Akt-positive tumor cells was determined using the Kruskal–Wallis test. Overall survival curves were estimated using the Kaplan–Meier method, and any differences in the survival curves were compared by the log-rank test. Western blot results were digitized with Image Master software from Amersham Biosciences.

## Results

### Immunohistochemical analysis of FAK

**Figure 1:**
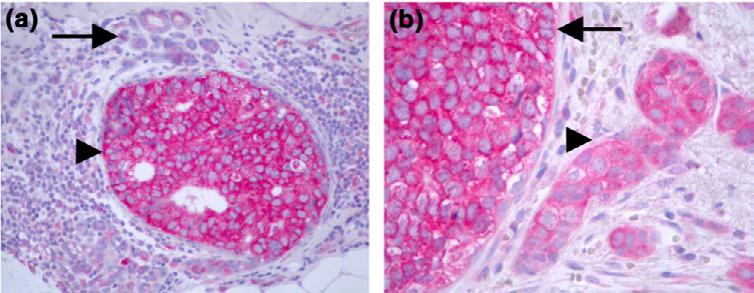
Immunohistochemistry for focal adhesion kinase (FAK) in ductal carcinoma *in situ*, invasive breast carcinonoma and normal breast tissue. **(a) **Strongest FAK expression was frequently seen in ductal carcinoma *in situ *(arrowhead). Notice the missing staining in normal breast tissue (arrow). **(b) **Invasive breast carcinoma cases (arrowhead) exhibited an equal or less intense FAK immunostaining then adjacent ductal carcinoma *in situ *(arrow) in all but one case. Original magnification, × 400.

Staining results were obtained from all 162 cases. In normal breast epithelium, a weak to missing FAK expression was detected at the cell membrane and in the cytoplasm. Strong FAK overexpression was detected in 30 patients (18.5%), intermediate staining in 119 patients (73.5%) and negative staining in 13 patients (8%). Higher FAK expression was significantly associated with a poorer differentiation grade. No statistically significant differences were found among cases with different tumor sizes or different tumor types (Table 1). The staining intensity of an adjacent *in situ *component was equal to or stronger than that of the invasive tumor, with only one sample exhibiting a weaker FAK staining in the *in situ *component than in the corresponding invasive carcinoma (Fig. 1).

### Correlation between FAK expression and survival

The impact of FAK expression and clinicopathological features on patient survival was assessed using univariate Kaplan–Meier survival analysis. Except for histological grading (*P *= 0.005), ER status (*P *= 0.0033) and activation of Akt (*P *= 0.0383), none of the other investigated parameters – including FAK expression (irrespective of ER expression) or HER-2/neu status – proved to be of prognostic significance in the node-negative breast cancers examined.

### Correlation of FAK protein expression with HER-2/neu status, ER status and phospho-Src Tyr-215 and phospho-Src Tyr-416 overexpression

A total of 159 tumors were analyzed for HER-2/neu protein expression: 59 tumors (37.1%) were classified as negative (0), 56 tumors (35.2%) as negative (1+), 21 tumors (13.2%) as weakly positive (2+) and 23 tumors (14.5%) as strongly positive (3+). Tumors with a score of 2+ and 3+ were defined as Her-2/neu-positive. A total 51.7% of tumors classified as strongly FAK-positive exhibited Her-2/neu protein overexpression, but intermediate classified tumors totaled only 24.5% of Her-2/neu overexpressing tumors. All of the FAK-negative tumors were Her-2/neu-negative. The ER status was obtained from 152 tumors. Ninety tumors (59.2%) showed a significant ER expression whereas 62 tumors (40.8%) were classified as negative.

**Figure 2:**
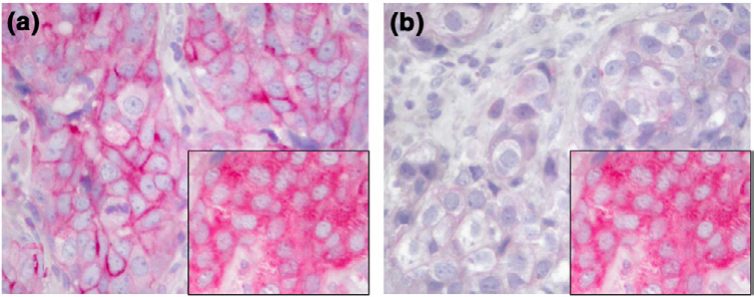
Representative immunohistochemical staining of a Her-2/neu and focal adhesion kinase (FAK)-overexpressing tumor for phospho-Src Tyr-215 and phospho-Src Tyr-416. Light micrograph displaying **(a) **strong phospho-Src Tyr-215 and **(b) **missing phospho-Src Tyr-416 staining in serial sections of a Her-2/neu-overexpressing and FAK-overexpressing invasive breast cancer sample as analyzed by immunohistochemistry. Inset: immunohistochemical staining showing FAK overexpression of the same sample. Original magnification, × 400.

**Figure 3:**
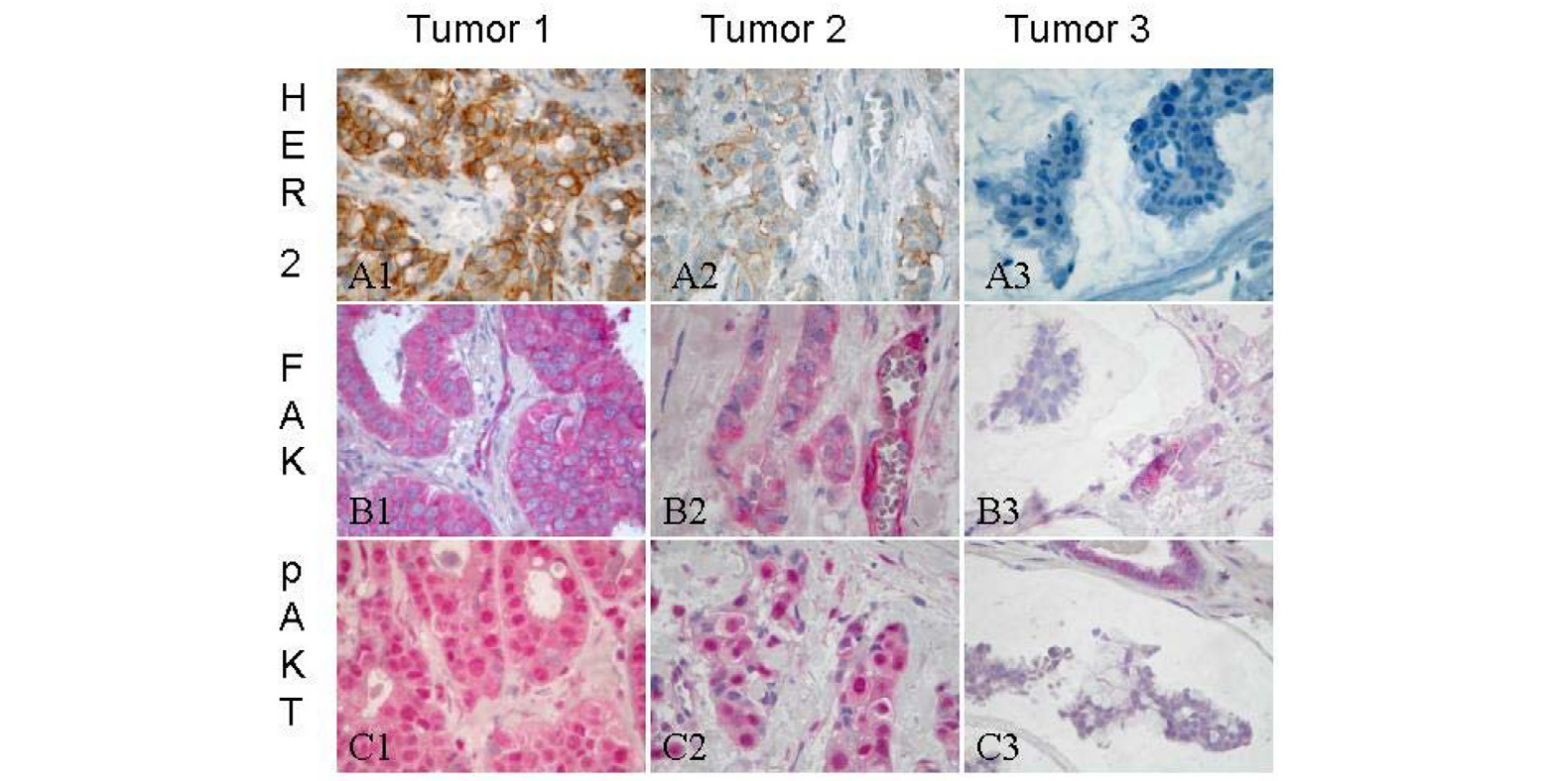
Representative immunohistochemical staining of serial sections of three tumors for phospho-Akt, focal adhesion kinase (FAK) and HER2. **(A1)–(A3) **HER2: tumors with strong HER2 staining intensity (DAKO score 3+) (A1), weak HER2 staining (DAKO score 1+) (A2) and missing HER2 staining (DAKO score 0) (A3). **(B1)–(B3) **Tumors showing strong HER2 immunostaining (A1) more frequently exhibited strong FAK immunostaining (B1), whereas tumors expressing weak or no HER2 immunostaining were more often associated with weak or no FAK immunostaining (B2, B3). **(C1)–(C3) **Phospho-Akt: the percentage of phospho-Akt positively stained tumor cells ranged from more than 80% (C1) to 45% (C2) or was lower than 45% (C3). Higher HER2 (A1) and FAK (B1) staining intensites are correlated with higher percentage of phospho-Akt positively stained tumor cells (C1, C2). Original magnification, × 200.

Statistical analysis revealed a significant relationship of elevated FAK expression (*P *= 0.001) with Her-2/neu overexpression (2+ and 3+; Table 3) but not with the ER status. Phospho-Src Tyr-215 staining was obtained from 137 patients and phospho-Src Tyr-416 staining was obtained from 140 samples. Normal breast tissue exhibited no phospho-Src Tyr-215 immunostaining. Membranous phospho-Src Tyr-416 immunostaining was noticed in sporadical epithelial cells. Myoepithelial cells lacked both phospho-Src Tyr-215 and phospho-Src Tyr-416 immunostaining. Tumor tissue of the remaining samples was not available due to lack of paraffin material. Statistical analysis revealed a significant direct correlation of FAK overexpression with phospho-Src Tyr-215 staining results (*P *= 0.021) but not with phospho-Src Tyr-416 staining scores (Table 3). On the other hand, positive phospho-Src Tyr-215 staining was significantly associated with HER2 overexpression (*P *= 0.011) whereas phospho-Src Tyr-416 was not. Representative immunohistochemical stainings are shown in Figs 2 and 3.

### Correlation between FAK expression and Akt/ERK activation

Normal breast tissue revealed mostly a cytoplasmatic Akt-staining pattern, with a low mean percentage of positive nuclear stained cells in normal breast tissue of 28%. Prominent nuclear and partly cytoplasmatic phospho-Akt immunoreactivity was noticed in tumor cells. Staining heterogeneity was occasionally apparent at the invasive tumor front. The percentage of phospho-AKT-positive nuclei in the tumor samples ranged from 0% to 85%. Tumors with more than 45% of positive nuclei were classified as phospho-Akt-positive.

Erk1/2 immunohistochemistry of tumor samples revealed strong nuclear immunostaining and partly cytoplasmatic immunostaining, while non-neoplastic tissue only occasionally revealed a weak staining. Similar to phospho-Akt, heterogeneous immunostaining for phospho-ERK1/2 was detected at the invasive tumor front. Phospho-Akt-positive classified tumors exhibited stronger FAK immunostaining intensities more frequently (*P *< 0.001) than phospho-Akt-negative classified tumors.

No correlation was observed between different FAK immunostaining scores and phospho-ERK1/2 immunostaining scores (*P *= 0.108) (Table 3). Statistical analysis revealed a significant direct correlation of different FAK immunostaining intensities with the percentage of phospho-Akt-positive stained tumor cells (*P *< 0.001).

### Correlation between HER2 protein overexpression and phosphorylation of FAK at Tyr-861 in western blot analysis

**Figure 4:**
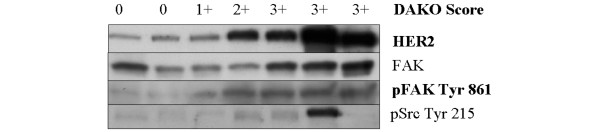
Upregulation of tyrosine phosphorylation of focal adhesion kinase (FAK) at tyrosine 861 in HER2-overexpressing breast tumors. Western blot analysis: lysates of seven breast cancer samples were analyzed by immunoblotting using antibodies that recognize total FAK and distinct tyrosine sites on Src and FAK kinases. Tumor samples were put in order according to the HER2 expression status (HerceptTest™) and the blot was probed with anti-HER2 antibody to analyze the exact amount of HER2 protein.

We next analyzed the relationship of HER2 overexpression with the levels of phospho-Src Tyr-215, phospho-FAK Tyr-861 and total FAK using seven samples of fresh-frozen breast cancer tissue. Of the seven samples analyzed, four tumors with high HER2 levels exhibited increased levels of phosphorylation of FAK on tyrosine 861 compared with tumors without HER2 overexpression (*P *= 0.01; Fig. 4). An increase of total FAK and phospho-Src Tyr-215 was noticed within the HER2 overexpressing tumors but reached not statistical significance. These results provide evidence that the HER/FAK Tyr-861 pathway is activated in human breast cancer.

## Discussion

Several studies have demonstrated upregulation of FAK expression in malignant tumors, including breast cancer, but the prognostic value of FAK expression in human cancer remains unclear. From the three studies that have previously analyzed this, one study denies the prognostic value of FAK in adenocarcinomas of the colon [16], whereas the other two studies have provided evidence for the prognostic value of immunohistochemically detected FAK expression in esophageal squamous carcinoma and in hepatocellular carcinoma, respectively [15,17]. The present study is the first to clarify the prognostic relevance of FAK expression in a cohort of 162 invasive node-negative breast cancer cases with long-term follow-up. Our findings demonstrate that FAK overexpression does not predict patient outcome in this setting; although, similar to esophageal squamous cancers, levels of FAK expression were strongly correlated with poorer tumor differentiation [17].

FAK is a nonreceptor protein kinase involved in integrin-mediated signaling that has a profound impact on cell proliferation, survival and migration. One downstream target is the PI3K/Akt signaling pathway. In the present study we found a significant association of elevated FAK expression with Akt phosphorylation in support of the notion that Akt may be a downstream target of FAK-mediated signaling [27]. However, while Akt activation is associated with a worse prognosis in our study cohort, FAK expression levels are not. This may be due to the central role of Akt in tumorigenesis, where a number of stimuli and pathways besides FAK contribute to the common end point of increased Akt activity.

Akt, also known as protein kinase B, is a serine/threonine protein kinase that has been shown to regulate cell survival signals in response to a diversity of signals generated by growth factors, cytokines and oncogenic ras. Alternative activation mechanisms for Akt besides growth factor-mediated PI3K stimulation are mutational inactivations of PTEN or activation via integrin-linked kinase [28-30]. The difference in prognostic relevance between Akt and FAK may therefore not be surprising. Several studies have demonstrated upregulation of FAK in human cancer including breast cancer and have suggested that FAK overexpression is an early event in tumorigenesis [13,14]. Our data support this hypothesis as all (but one) of the *in situ *carcinomas adjacent to the invasive cancer exhibited FAK overexpression. Interestingly, in every cancer specimen analyzed the adjacent preinvasive component exhibited an equal or even higher FAK immunostaining than the corresponding invasive carcinoma.

In recent studies, Vadlamudi and colleagues utilized human breast cancer cell lines *in vitro *to establish a novel signaling pathway involving HER2, phospho-Src Tyr-215 and phospho-FAK Tyr-861 leading to increased cellular motility [18,19]. The authors showed that heregulin-induced HER2 activation resulted in phosphorylation of FAK at tyrosine 861, while six breast tumor samples exhibited increased level of phospho-Src Tyr-215 in HER2 neu-overexpressing breast cancers. From these *in vitro *data it was tempting to hypothesize that HER2 influences metastasis of breast cancer *in vivo *via a similar pathway.

To test for the existence of this novel pathway in a large cohort of human breast cancer tissue we evaluated total FAK protein and phospho-Src Tyr-215 expression as well as phospho-Src Tyr-416 expression by immunohistochemistry. Additionally, we investigated the levels of phospho-FAK Tyr-861, phospho-Src Tyr-215 and Her-2/neu in a set of seven of freshly frozen breast cancer tissues using western blot analysis. Our findings support the hypothesis that HER2-overexpressing tumors exhibit higher levels of phospho-FAK Tyr-861 but also of total FAK levels. This is also supported by the immunohistochemical data where Her-2/neu protein expression levels coincide with FAK protein expression. Interestingly, increased levels of phospho-Src Tyr-215 but not of phospho-Src Tyr-416 were significantly associated with HER2 overexpression, supporting the role of an activatory Src Tyr-215 phosphorylation as an intermediate between HER2 and FAK signaling.

Although Src comprises several phosphorylation sites, phosphorylation of tyrosine 215 and not tyrosine 416, the 'classical' activatory phosphorylation site, appears to be essential for FAK activation via HER2 [19]. Our data confirm the specificity of this pathway as src phosphorylation at tyrosine 416 was not associated neither with HER2 overexpression. The present study was designed to investigate this novel signaling pathway *in vivo *via an immunohistochemical approach allowing the determination of HER2 and FAK protein expression.

A recent study identified frequent polysomic patterns for chromosome 1, chromosome 8 and chromosome 17 that are indicative for increased tumor malignancy in breast cancer [31]. As the focal adhesion kinase and the Her-2/neu gene are located on chromosome 8 and chromosome 17, respectively, one might conclude that such a polysomic pattern causes a combined HER2/FAK protein overexpression. However, the identification of potential underlying causative genetic alterations should be a topic of further investigation.

Our results propose that among the many biological effects of Her-2/neu overexpression the activation of the Akt survival pathway via the FAK Tyr-861–Src Tyr-215 pathway might contribute to the more aggressive behavior of Her-2/neu-overexpressing breast cancers.

**Figure 5:**
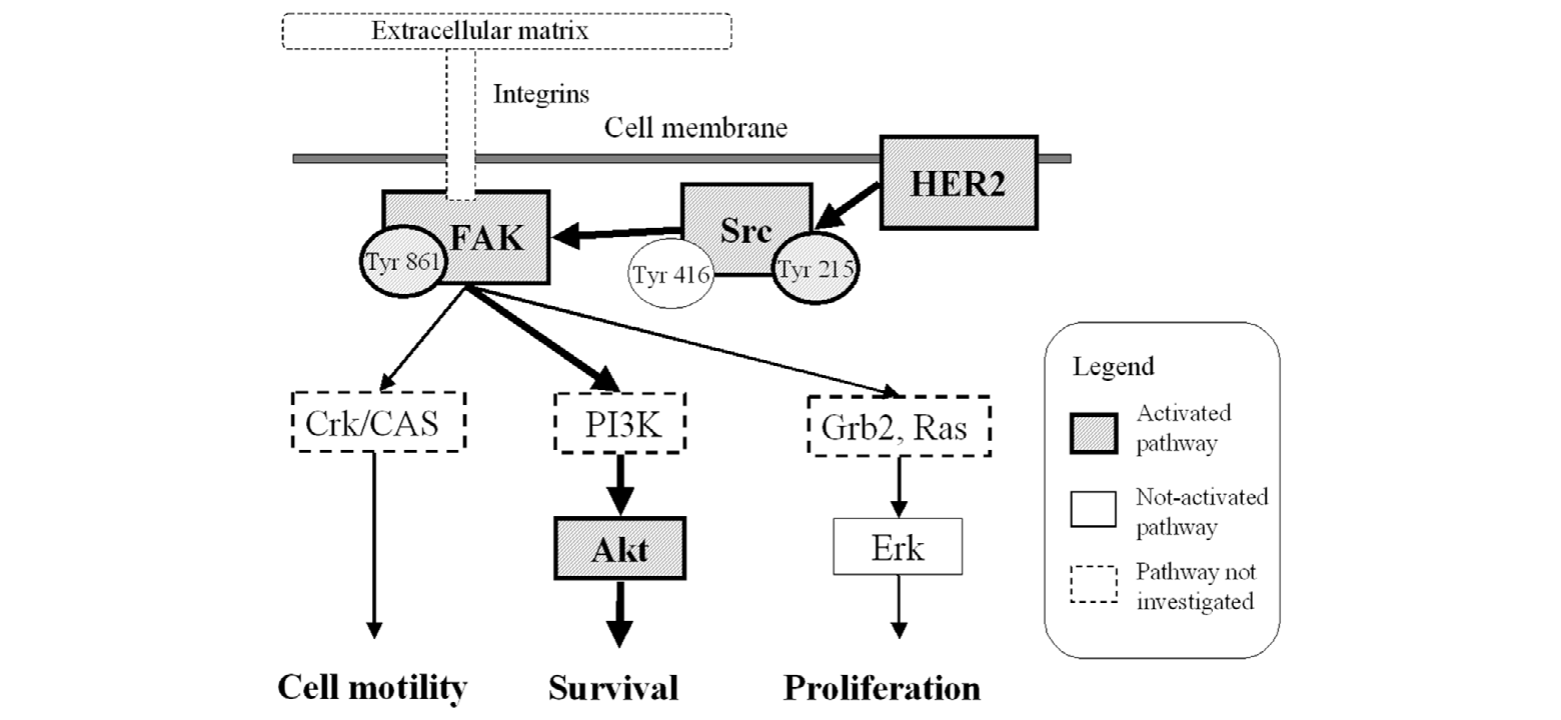
Scheme depicting signalling transduction in breast cancer involving HER2, Src, focal adhesion kinase (FAK), phosphatidylinositol 3-kinase (PI3K), Akt and Erk.

In summary, FAK overexpression is not a prognostic marker in this series of 162 node-negative breast cancers. This might be due to the composition of our cohort, which mainly contains early-stage cancers without lymph node metastasis. Recent studies demonstrated a higher level of FAK expression in colorectal liver metastasis than in the primary tumor, and provided evidence for an upregulation of FAK protein in hepatocellular cancers with portal invasion [15]. Nevertheless, FAK protein overexpression coincides with Her-2/neu overexpression and may mediate HER2 signaling via Src, resulting in PI3K/Akt activation. These data might contribute to the understanding of how Her-2/neu overexpression in human breast cancer affects tumor malignancy and metastasis (Fig. 5). Owing to the relatively small number of breast cancer samples exhibiting FAK protein overexpression in this cohort, however, these promising results need to be confirmed in larger cohorts.

## Abbreviations

ER = estrogen receptor; ERK = extracellular regulated kinase; FAK = focal adhesion kinase; PI3K = phosphotidylinositol-3-kinase.

## Competing interests

There has been no prior publication of the content of this study. This paper has not been submitted to any other journal. No financial relationship exists that might lead to conflict concerning the content of this study. The paper has been read and approved by all of the authors. The authors declare that they have no competing interests.

## Authors' contributions

KJS carried out the primary immunohistochemical staining evaluation, performed statistical analysis and wrote the article. FG, FO and JW carried out the secondary immunohistochemical staining evaluation to rule out interindividual observer discrepancies. RC and RK provided the clinical data. BL contributed substantially to the conception and design of the study and to the drafting of the manuscript. KWS performed critical revision of the manuscript for important intellectual content. HAB provided administrative, technical and material support and supervision, and analyzed western blot data. All authors read and approved the final manuscript.

## Figures and Tables

**Table 1:** Clinicopathological data and focal adhesion kinase expression in 162 node-negative breast cancers

	Focal adhesion kinase expression			*P *value*
		
	Negative	Strong	Intermediate	
Total adenocarcinoma	13 (8%)	30 (18.5%)	119 (73.5%)	
Tumor type				Not significant
Invasive ductal	5 (4.1%)	27 (22.5%)	88 (72.7%)	
Invasive lobular	3 (13%)	2 (8.7%)	18 (78.3%)	
Mucinous	5 (62.5%)	/	3 (37.5%)	
Tubulo/tubulo-lobular	/	/	5 (100%)	
Others	/	1 (16.7%)	5 (88.3%)	
Histological grade				*P *= 0.038*
Well differentiated	4 (11.4%)	3 (8.6%)	28 (80%)	
Moderately differentiated	6 (7%)	13 (15.1%)	67 (77.9%)	
Poorly differentiated	3 (7.3%)	14 (34.1%)	24 (58.5%)	
Tumor size				Not significant
pT1(a,b,c)	7 (7.2%)	21 (21.6%)	69 (71.1%)	
pT2	5 (7.9%)	9 (14.3%)	49 (77.8%)	
pT3 and pT4	1 (50%)		1 (50%)	

* *P *value of chi-squared analysis.

**Table 2:** Primary antibodies used for immunohistochemistry

Antibody		Dilution	Source
Phospho-Akt (1/2/3) Serin 473	Polyclonal	1:200	Cell Signaling Technology
Phospho-Akt (1/2/3) Serin 473	Polyclonal	1:2000	Santa Cruz Biotechnology
Focal adhesion kinase (C-20)	Polyclonal	1:100	Santa Cruz Biotechnology
Phospho-focal adhesion kinase tyrosine 861	Polyclonal	1:50	Santa Cruz Biotechnology
Phospho-Src tyrosine 215	Polyclonal	1:200	Sigma-Aldrich
Phospho-Src tyrosine 416	Polyclonal	1:200	Cell Signaling Technology
Phospho p44/42 Map kinase (Erk1/2) threonine 202;tyrosine 204	Monoclonal	1:100	Cell Signaling Technology
Estrogen receptor	Monoclonal	1:25	DAKO

**Table 3:** Correlation between focal adhesion kinase, HER2, estrogen receptor, phospho-Src Tyr-215 and phospho-Src Tyr-416, phospho-Akt and phospho-ERK1/2 immunohistochemistry

	Focal adhesion kinase expression			*P *value*
		
	Negative	Intermediate	Strong	
Her-2/neu status (*n *= 159)				0.001
Negative (0, 1+)	13	88	14	
Positive (2+, 3+)	0	29	15	
Estrogen receptor status (*n *= 152)				Not significant
Negative	4	48	10	
Positive	6	67	17	
Phospho-Src Tyr-215 (*n *= 137)				0.021
Negative	9	87	17	
Positive		15	9	
Phospho-Src Tyr-416 (*n *= 140)				Not significant
Negative	8	86	21	
Positive		20	5	
Phospho-Akt^a ^(*n *= 162)				< 0.001
Negative	7	10	1	
Positive	6	109	29	
Phospho-ERK1/2 (*n *= 161)				Not significant
Negative	5	28	9	
Intermediate	8	74	13	
Strong		16	8	

* *P *value of chi-squared analysis.

^a^Negative, percentage of phospho-Akt-positive cells ≤ 45%; positive, percentage of phospho-Akt-positive cells > 45%.
